# Drug-induced comorbidities in patients with sarcoidosis

**DOI:** 10.1097/MCP.0000000000000889

**Published:** 2022-07-18

**Authors:** Marjolein Drent, Naomi T. Jessurun, Petal A. Wijnen, Otto Bekers, Aalt Bast

**Affiliations:** a Department of Pharmacology and Toxicology, Faculty of Health, Medicine and Life Science, Maastricht University, Maastricht; b ILD Center of Excellence, Department of Respiratory Medicine, St. Antonius Hospital, Nieuwegein; c ILD Care Foundation Research Team, Ede; d Netherlands Pharmacovigilance Centre Lareb, ‘s-Hertogenbosch; e Central Diagnostic Laboratory, Department of Clinical Chemistry, MUMC, Maastricht, The Netherlands

**Keywords:** adverse drug reactions, comorbidity, drug-induced comorbidity, glucocorticoids, pharmacogenomic testing, sarcoidosis, sarcoidosis-associated morbidity, therapeutic drug monitoring, treatment

## Abstract

Sarcoidosis is a chronic multisystemic inflammatory disease of unknown aetiology with a wide range of highly variable clinical manifestations and unpredictable disease course. Sarcoidosis patients may present with specific organ-related symptoms involving functional impairments, and less specific symptoms. The decision whether and when to treat a sarcoidosis patient with pharmacotherapy depends on two major factors: risk of organ failure and/or death and impairment of quality of life. This decision is complex and not standardized.

**Recent findings:**

Glucocorticoids (GCs) are recommended as initial treatment, when needed. Subsequent GC-sparing alternatives frequently follow. Comorbidities or adverse drug reactions (ADRs) from drugs used in sarcoidosis treatment are sometimes very hard to differentiate from symptoms associated with the disease itself, which may cause diagnostic dilemmas. An ideal approach to minimalize ADRs would involve genetic screening prior to prescribing certain ‘high-risk drugs’ and therapeutic drug monitoring during treatment. Pharmacogenomic testing aims to guide appropriate selection of medicines, with the potential of reducing unnecessary polypharmacy while improving clinical outcomes.

**Summary:**

A multidisciplinary approach to the management of sarcoidosis may avoid unnecessary ADRs. It is important to consider the possibility of drug-induced damage in sarcoidosis, especially if the clinical situation deteriorates after the introduction of a particular drug.

## INTRODUCTION

Sarcoidosis is a multisystemic inflammatory disease of unknown cause with a wide range of clinical manifestations. A dysregulated immune response to certain environmental antigens is thought to result in sustained granulomatous inflammation and failure to clear the offending antigens [1^▪^]. Other conditions, such as infectious disorders and cancer, have also been shown to be associated with a granulomatous reaction, mimicking sarcoidosis [2]. In addition, certain drugs may also induce sarcoid-like reactions indistinguishable from sarcoidosis. Strictly speaking, the latter reactions are beyond the scope of this review as they do not constitute drug-induced comorbidity of sarcoidosis, but drug-induced sarcoidosis. The list of drugs associated with these sarcoid-like reactions includes antiretroviral therapy, tumour necrosis factor alpha (TNF-α) inhibitors, interferon therapy, and immune checkpoint inhibitors [3,4]. Pharmacotherapy for sarcoidosis itself or for the treatment of certain comorbidities may also be associated with drug-induced damage. The clinical manifestations and/or symptoms of these iatrogenic conditions may be difficult to distinguish from sarcoidosis-associated symptoms. Timing, the pattern of illness, the results of investigations, and rechallenging can help attribute causality to a suspected adverse drug reaction (ADR) [5]. Nowadays pharmacogenomic (PGx) testing to identify patients with genetic variants that put them at risk of ADRs and/or sub-optimal therapy is rapidly gaining ground [6^▪^]. Predicting a patient's response to particular drugs could support the safe management of medications and reduce morbidity and hospitalization, especially in case of polypharmacy [6^▪^,^7^,^8^]. However, everything starts with recognizing and considering ADRs early on. This review discusses the ADRs of sarcoidosis treatment and comorbidities and compares them with similar symptoms of sarcoidosis itself. It also discusses the predictive role of PGx in explaining or predicting the effect, whether the drug effect is suspected to be positive or negative. 

**Box 1:**
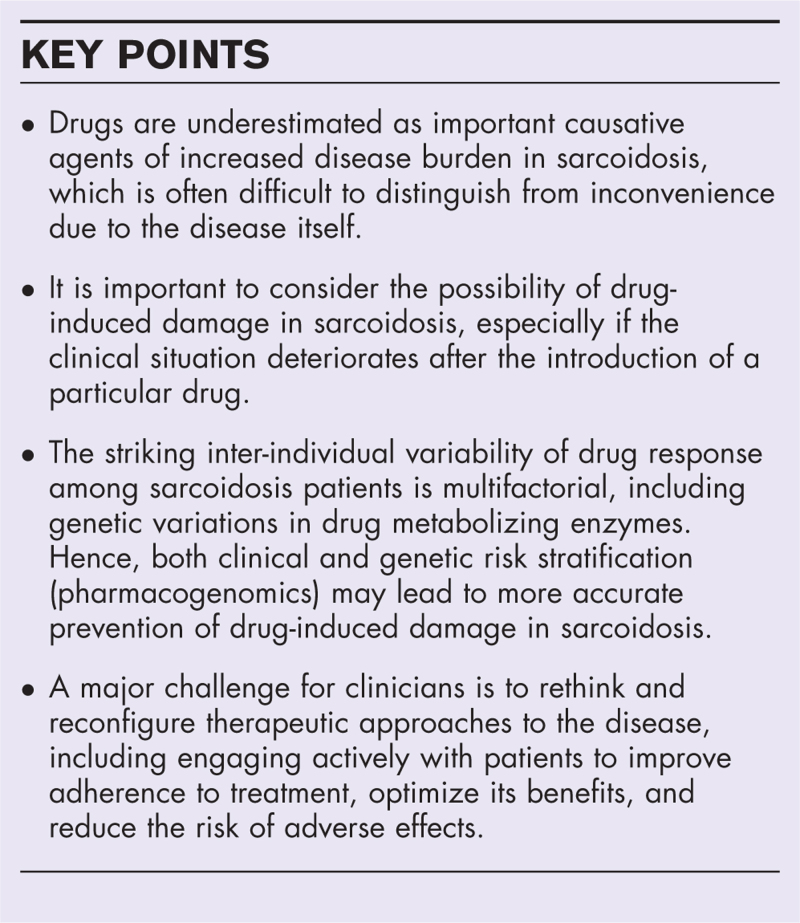
no caption available

## CLINICAL IMPACT OF PHARMACOLOGIC SARCOIDOSIS TREATMENT

The major goals in treating sarcoidosis are lowering the morbidity and mortality risk and/or improving quality of life (QoL) [9^▪^]. The drugs currently used to treat various manifestations of sarcoidosis – often immunosuppressive – are liable to cause ADRs and sometimes substantial co-morbidities. These co-morbidities further increase the burden of this disease, and affect the QoL [10,11,12^▪▪^]. The ADR profile associated with treatment of sarcoidosis varies greatly from one patient to the next, as does the efficacy of treatments, so a simple treatment algorithm that works for all patients is not feasible [9^▪^]. In general, there are three lines of therapy for sarcoidosis.

## FIRST-LINE TREATMENT

### Glucocorticoids

A recent consensus statement from sarcoidosis experts still endorses glucocorticoids (GCs) as the primary treatment for sarcoidosis, in view of their efficacy and ease of use (low cost and oral administration) [9^▪^]. Unfortunately, GCs are associated with a dose-dependent risk of serious ADRs, which tend to accumulate with long-term use (see also Table 1) [1^▪^,^13^–^17^].

**Table 1:** Summary of clinically relevant adverse drug reactions (ADRs) of glucocorticoids, low-dose methotrexate, azathioprine, hydroxychloroquine and infliximab, by organ system affected, as well as sarcoidosis-associated morbidity (SAM) [1^▪^,^13^–^17^]

Organ system affected	Glucocorticoid ADRs	Methotrexate ADRs	Azathioprine ARDs	Hydroxychloroquine ADRs	Infliximab ARDs	Sarcoidosis-associated morbidity (SAM)
Cardiovascular	Hypertension Coronary heart disease Ischaemic heart disease Heart failure	Pericardial serositis	Tachycardia	Arrhythmia Ventricular tachycardia Cardiomyopathy Bundle branch block/atrioventricular heart block Myocarditis	Tachycardia Palpitations Hypotension Hypertension Flushing	Palpitations Heart rhythm disturbances Chest pains Heart failure Syncope
Dermatologic	Dermatoprosis Skin atrophy Ecchymosis Purpura Erosions Striae Delayed wound healing Easy bruising Acne Hirsutism Hair loss	Oral ulcers Alopecia Rash Anaphylactic reactions Photosensitivity Vasculitis Nodulosis	Skin rash Hair thinning or hair loss Alopecia Photosensitivity reactions	Skin rash Skin pigmentation Pruritus Hair loss Alopecia	Skin rash	Macular or plaque skin lesions, especially over the face and hands, and involving tattoos Alopecia Vitiligo
Endocrine and metabolic	Hyperglycaemia Diabetes mellitus Dyslipidaemia Weight gain Cushingoid features Growth suppression Adrenal suppression Secondary hyperparathyroidism		Fever Weight loss	Fever Anorexia Weight gain/loss Hyperglycaemia Porphyria	Dyslipidaemia	Fever Hypothalamic-pituitary infiltration by sarcoid granulomata Hypogonadism Hypercalcemia
Gastrointestinal	Gastritis Peptic ulcer Gastrointestinal bleeding Visceral perforation Pancreatitis	Loss of appetite Nausea Vomiting Diarrhoea Gastrointestinal bleeding Complications of ulcers	Loss of appetite Nausea Pancreatitis Diarrhoea Obstipation Vomiting	Vomiting Diarrhoea Abdominal pain	Abdominal pain Nausea	Gastrointestinal involvement Diarrhoea
Hepatic	Hepatic steatosis	Elevated liver enzymes Fibrosis Cirrhosis	Cholestasis	Elevated liver enzymes Fulminant hepatitis	Abnormal hepatic function Increased transaminases	Liver involvement Elevated liver enzymes Pruritus Jaundice Fibrosis Ascites
Immunologic or haematologic	Suppression of cell-mediated immunity	Suppression of cell-mediated immunity Bone-marrow depression	Hypersensitivity reactions Stevens-Johnson syndrome Toxic epidermal necrolysis	Agranulocytosis Bone-marrow depression (Aplastic) anaemia Leukopenia Thrombocytopenia	Anaemia Leukopenia Neutropenia Thrombocytopenia	Splenomegaly Thrombocytopenia
Infectious disorders	Predisposition to infections Reactivation of latent infections	Opportunistic infections	Decreased resistance to infections		Opportunistic infections Viral infections Bacterial infections Tuberculosis Fungal infections	Decreased resistance to infections
Musculoskeletal	Osteoporosis Avascular necrosis of femoral head Myopathy	Osteopathy	Joint or muscle pain	Muscle weakness Myopathy Atrophy Joint or muscle pain		Muscle weakness/atrophy Myopathy Adjacent joints and bones can be involved
Neurologic or neuropsychiatric	Fatigue Cognitive impairment Mood alteration Depression Euphoria Irritability Akathisia Anxiety Psychosis Dementia Delirium	Fatigue Cognitive impairment Mood alteration Dizziness Headache Vertigo	Fatigue General weakness Headache Itching	Fatigue Headache Dizziness Nerve deafness Vertigo Tinnitus Emotional lability Depressive symptoms Psychosis Suicidal behaviour Nightmares	Fatigue Depression Insomnia Headache Dizziness Vertigo Paraesthesia	Fatigue Cognitive impairment Mood alteration Depressive symptoms Headaches Seizures Cranial nerve deficits Anxiety Focal peripheral neuropathies Small fibre neuropathy
Ophthalmologic	Posterior subcapsular cataract Increased intraocular pressure Glaucoma Ptosis Mydriasis Opportunistic ocular infections Central serous chorioretinopathy	Conjunctivitis Blurred vision Photophobia Blepharitis Decreased tear secretion reflex Peri-orbital oedema Nonarthritic ischemic optic neuropathy		Blurred vision Retinopathy Maculopathies Macular degeneration	Conjunctivitis	Painful conjunctivitis Vision loss Photophobia Hyperaemia Teary eyes Floaters Blurring, gritty eyes Uveitis Glaucoma
Renal		Renal insufficiency (only with preexisting, severely impaired renal function)				Granulomatous interstitial nephritis Nephrocalcinosis Haematuria Nephrolithiasis
Respiratory	Obstructive sleep apnoea	Interstitial pneumonitis Hypersensitivity pneumonitis Pneumocystis Jirovecii pneumonia (PJP)	Cough Troubled breathing with movement Interstitial pneumonitis	Allergic reactions Bronchospasms Hypersensitivity	Allergic respiratory symptoms Upper respiratory tract infection	Obstructive sleep apnoea Various pulmonary manifestations Endstage pulmonary fibrosis
Urogenital		Abortion Malformation Defective oogenesis and spermatogenesis Gynaecomastia	Painful or difficult urination Dark urine			Azoospermia Bladder dysfunction Multiple sarcoidosis localizations female genitalia Granuloma in the breast

ADR, adverse drug reaction.

Moreover, no noticeable benefits in disease outcomes are observed with higher versus lower doses, particularly in maintenance therapy. The most frequent self-reported ADRs related to GC use are weight gain and increased appetite [18]. This increases the risk of other co-morbidities such as diabetes, exercise limitations, obstructive sleep apnoea, and fatigue. Moreover, GC use in immune-mediated diseases, including sarcoidosis, increases the risk of cardiovascular disease [19]. Of note, fatigue is one of the most devastating, unexplained symptoms of sarcoidosis itself, but it can be exacerbated by its treatment, and can also be associated with withdrawal of GCs [20,21]. Extensive use of GCs has also been linked to increased rates of nonsarcoidosis-related emergency department visits compared to patients with lower cumulative GC exposure [22].

**FIGURE 1:**
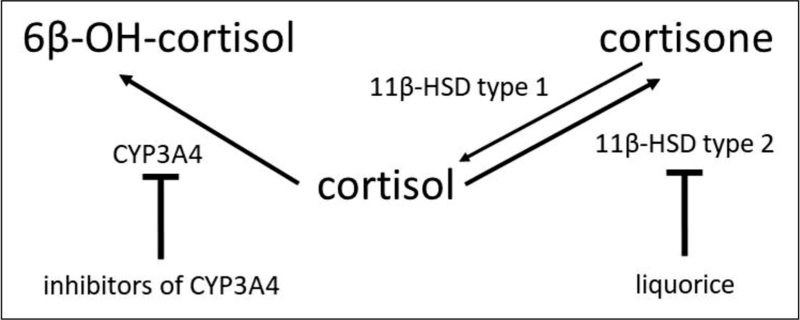
Agents influencing the biotransformation of cortisol. Liquorice inhibits renal 11β-hydroxysteroid dehydrogenase type 2 (11β-HSD type2), resulting in higher levels of cortisol, which then binds to and activates mineralocorticoid receptors (as does aldosterone), increasing blood pressure. The ratio 6b-hydroxy cortisol vs. cortisol is used as a marker of cytochrome P450 3A4 (CYP3A4) activity. Inhibition of this iso-enzyme by other drugs or food such as grapefruit juice will influence the biotransformation of cortisol.

It is of great clinical importance to try and minimize the ADRs of GCs [1^▪^,9^▪^]. Obviously, there is interindividual variability of the metabolism of GCs and this can have a substantial impact upon their phenotypic effects. In recent years, several studies have clarified the mechanism of action of GCs at the molecular level, and the role of genetic variants in their efficacy [23]. Drug metabolizing enzymes of the cytochrome P450 3A4 (CYP3A) subfamily play a certain role in GC metabolism (see Fig. 1) [24,25^▪^]. Since GCs are metabolized by CYP3A4, various CYP3A4 inhibitors may reduce GC degradation, increasing its accumulation, and may induce comorbidities such as iatrogenic Cushing's syndrome [26]. To date, P450 3A4 is known to metabolize more than 50% of clinically used drugs. In addition, inhibitors of 11-beta-hydroxy-steroid dehydrogenase may increase the effect of GCs. In contrast, patients on CYP3A4-inducing drugs who also take GCs may require greater GC doses to achieve the intended treatment effect. In this situation, choosing an alternative drug, if possible, may be advisable [27]. As there is considerable variation in individual needs regarding the degree of GC replacement, the balance between over- and under-replacement of GCs is a significant clinical challenge [28]. Strikingly, the presence of *CYP* polymorphisms has also turned out to be a substantial susceptibility risk factor in the development of a GC withdrawal syndrome, by influencing the cortisol metabolism [25^▪^,^29^]. There is clear interindividual variability of the metabolism of cortisol, and this can have a profound impact upon the phenotypic effects of GCs [29]. Polymorphisms in the cytokine regulatory regions might therefore result in variable levels of inflammation and response to GCs (Table 1) [18].

Unfortunately, hardly any genetic markers have so far been identified as predictors of efficacy or ADRs that could be useful for the development of dosing guidelines [30]. Gathering information about the phenotypes in relation to genotypes (including PGx) would help to find out which individuals might be predisposed to certain ADRs and co-morbidities. Genotyping prior to drug prescription may be clinically relevant for predicting the response to GCs, as well as for the prevention of ADRs and the development of a withdrawal syndrome. This information should guide a more personalized drug prescription. Besides reducing GC doses by combining them with immunosuppressive drugs, new strategies for optimizing treatment need to be explored, including lifestyle changes, physical therapy, and dietary guidance [1^▪^]. Thus, for patients requiring long-term suppression of inflammation to prevent irreversible organ damage or intolerable symptoms, early institution of GC-sparing antisarcoidosis agents is generally recommended, either alone as a first-line treatment or combined with a rapid reduction in GC doses. In clinical practice, however, high doses of GCs are still often used, causing substantial co-morbidity.

## SECOND-LINE TREATMENT

### Methotrexate

Second-line agents are useful especially if a patient experiences undesirable ADRs or is resistant to GCs. GC-sparing alternatives for sarcoidosis include methotrexate (MTX), azathioprine (AZA), leflunomide, mycophenolate mofetil (MMF) and hydroxychloroquine (HCQ) [1^▪^,9^▪^,^10^].

MTX, an anti-inflammatory agent and immunomodulator, can cause many ADRs, related to its mechanism of action (see Table 1) [31]. Although MTX is known to be associated with many ADRs, ADR information sources unfortunately do not distinguish between high and low dose administration.

Low dose MTX (LD MTX) toxicity and efficacy is associated with a number of polymorphic enzymes, and testing for variants was found to predict response to and safety of LD MTX treatment [32,33]. Moreover, when a patient develops an ADR after starting LD MTX treatment, one should consider testing the C677T and A1298C variants of the methylenetetrahydrofolate reductase (MTHFR) gene, which is involved in MTX metabolism [32]. LD MTX has been shown to be a successful treatment option for a wide array of severe sarcoidosis manifestations and an important agent – often regarded as the second-line treatment of first choice – in the management of sarcoidosis [31–33]. Its long-term safety and efficacy in sarcoidosis remain unclear, however, hepatotoxicity is an important risk. MTX also causes several nonspecific abnormal laboratory findings including moderate leucocytosis, mild eosinophilia, and elevated transaminase levels (e.g. lactate dehydrogenase (LDH)). New or progressive imaging features such as PET-scan activity must be differentiated from pre-existent sarcoidosis-associated features. A retrospective study of sarcoidosis patients found very few hepatic or hematologic complications, so the authors considered LD MTX (7.5–25 mg/week) to be a safe and effective treatment [34].

Safety considerations also include the co-administration of other drugs. However, the clinical importance of the interaction between LD MTX and nonsteroidal anti-inflammatory agents (NSAIDs), penicillin and proton pump inhibitors (PPIs) cannot be substantiated and is only rarely clinically relevant [35]. Since MTX is a folate analogue that antagonizes the enzyme dihydrofolate reductase, as well as disrupting DNA synthesis, DNA damage repair, and cellular replication, folate supplementation is strongly recommended with MTX therapy [31,36]. As stated by Beduoi *et al.*[37^▪^], some of the MTX-associated side effects can be related to its mechanism of action (see Table 2).

**Table 2:** Various mechanisms of low-dose methotrexate dampening the inflammatory response [37^▪^]

1. Inhibition of purine and pyrimidine synthesis
2. Promotion of adenosine release with adenosine-mediated suppression of inflammation and of high-mobility group box chromosomal protein 1 (HMGB1) thus inhibiting Toll-like receptor (TLR) activation
3. Suppression of transmethylation reactions and polyamine accumulation
4. Reduced production of matrix metalloproteinases (MMPs), which play a role in tissue remodelling. Reduced synthesis of the inflammatory mediator prostaglandin E2 (PGE2) and of cytokine expression
5. Suppression of the activation of the transduction pathways Janus kinase/signal transducers and activators of transcription (JAK/STAT) and nuclear factor-κB (NF-κB); hence inhibition of the upregulation of multiple pro-inflammatory cytokines

Gavrysyuk *et al.*[38^▪^] showed that in patients with pulmonary sarcoidosis, LD MTX monotherapy does not differ significantly from methylprednisolone monotherapy in efficacy levels and ADR occurrence. Increasing the MTX dose from 10 to 15 mg/week accelerated regression rates of sarcoidosis, improved treatment efficacy, and did not change ADR frequency. Furthermore, there was a significant decrease in the incidence of treatment resistance and relapse rate. In line with this, a randomized controlled trial among patients with pulmonary sarcoidosis, is investigating the effectiveness and tolerability of MTX as first-line therapy compared with prednisone [39]. If this study also confirms the hypothesis that LD MTX is as effective as prednisone as a first-line treatment, with fewer ADRs, this will have a major clinical impact. This latter study will also gather additional information about the prevalence of ADRs, as there are a number of MTX-induced ADRs that mimic symptoms of sarcoidosis such as neuropsychiatric problems, fatigue, and various pulmonary manifestations.

### Hydroxychloroquine

Hydroxychloroquine, first introduced as an antimalarial medication, has been recommended/used in the treatment of sarcoidosis as well [1^▪^]. It has been particularly useful in cutaneous disease, hypercalcaemia, and some cases of neurosarcoidosis [11]. The mechanisms of action of hydroxychloroquine are varied; it can interfere with antigen presentation, prevent T-cell activation, inhibit toll-like receptor signalling, and reduce the production of inflammatory cytokines by T-cells and B-cells [40]. Hydroxychloroquine influences the levels of metoprolol as it inhibits its metabolism by competing for the same CYP enzyme, CYP2D6. Since antimalarial drugs are thought to interfere with medications that influence the QT interval, patients on hydroxychloroquine therapy who concurrently take such drugs for the treatment of cardiac comorbidities should also be monitored for the potential risk of cardiac arrhythmia. Previously, glucose-6-phosphate dehydrogenase (G6PD) deficiency, which causes susceptibility to the haemolytic effect of drugs as well as certain other agents, also appeared to be involved in the development of pulmonary and cardiac toxicity [41]. It was shown that G6PD deficiency contributes to cardiac dysfunction through increased susceptibility to oxidative injury. Drent *et al.*[43] reported the antioxidant action of carvedilol, which might prevent the effects of G6PD deficiency in combination with sarcoidosis [42]. As suggested by Jain *et al.*[44,45] inactivity of G6PD will prevent adequate formation of NADPH and thus hamper glutathione maintenance (GSH). Sarcoidosis has been suggested to trigger an oxidative stress response, as indicated by an increased activation of nuclear factor kappa B (NF-κB). This may also lead to a decrease in cytosolic GSH. Moreover, a decreased NADPH level in erythrocytes was found in a considerable percentage of female sarcoidosis patients. Increased utilization of reduced NADPH may possibly be involved in the inflammatory process triggered by oxidative stress in sarcoidosis [46]. Hence, in case of clinical deterioration after a sarcoidosis patient has started hydroxychloroquine, an ADR associated with a G6PD deficiency should be considered, especially if other causes are excluded. Gastrointestinal ADRs – although common – are generally mild and well tolerated [11]. The ADRs are summarized in Table 1.

### Azathioprine

Azathioprine (AZA), a purine antagonist, derives its anti-inflammatory effect mainly from reducing B- and T-cell proliferation. In the management of sarcoidosis, it is used as second-line treatment in case MTX is contraindicated (e.g. due to pregnancy) or has failed to produce a response [47]. In view of their slow time to clinical response, between 8 and 12 weeks, both these agents are not suitable for rapid induction of remission. In a retrospective unblinded uncontrolled cohort study, AZA and MTX yielded similar outcomes [47]. Reported ADRs are more or less comparable with those of MTX [16]. Most frequent ADRs include infections, gastro-intestinal complaints, hepatic function decline, pancreatitis, bone marrow depression, as well as fever and fatigue (see also Table 1). The latter symptoms can be hard to differentiate from sarcoidosis-associated symptoms. AZA is metabolized by thiopurine *S*-methyltransferase (TPMT), and patients with low TPMT levels can develop severe neutropenia. TPMT genotyping is advised before starting treatment with AZA to identify patients susceptible to toxicity [48]. Co-administration of drugs that influence TPMT or xanthine oxidase activity, such as allopurinol can increase AZA efficacy by increasing the 6-tioguanine (thioguanine) nucleotides (6-TGN) concentration. By reducing the 6-methyl mercaptopurine ribonucleotides (6-MMPR) metabolite concentration allopurinol reduces the risk of hepatotoxicity [49].

### Third-line treatment

#### Biologicals

Third-line treatment consists of biologicals (e.g. TNF-α inhibitors, particularly infliximab and adalimumab), and is currently reserved for patients suffering unacceptable side effects, or showing no or insufficient response to first- or second-line treatment, or severe threatening disease manifestation at baseline [1^▪^,9^▪^,^10^,^11^,^50^].

About 70% of patient show improvement or at least stabilization after third-line treatment [50,51]. Several studies have considered the possibility that the variability of its effects is caused by variants in genes involved in immune processes, inflammation, autophagy, and apoptosis. Two SNPs (rs1800629 and rs361525) located in the TNF-α promoter region seem to be involved in TNF expression and TNF inhibitor response, particularly in patients with rheumatological conditions and Crohn's disease [23,52]. The rs1800629 SNP is a −308G>A substitution and influences the regulation of TNF-α synthesis; in particular, −308A confers a major transcriptional activation and increases TNF-α production compared with the common −308G. AA and GA genotypes were found to be correlated with nonresponse to TNF inhibitor treatment [23,52]. In line with this, Wijnen *et al.*[53] found that sarcoidosis patients without the -308A variant allele (GG genotype) had a three-fold higher response to TNF inhibitors (adalimumab or infliximab).

The most frequently reported and clinically relevant ADRs include infections (see also Table 1). Research has shown that concomitant use of adjunctive immunosuppressants such as MTX reduces the risk of neutralizing antibodies and increases drug efficacy [54]. This underlines the importance of keeping in mind that drug levels are substantially influenced by concomitant use of certain other drugs.

## ADVERSE DRUG REACTIONS ASSOCIATED WITH PHARMACOTHERAPY OF COMORBIDITIES FOLLOWED BY CLINICAL DETERIORATION

Sarcoidosis patients are sometimes treated for comorbidities as well, which may cause ADRs and have an impact on QoL. This possibility should be considered if a sarcoidosis patient develops clinical-pathologic symptoms similar to those of sarcoidosis during treatment with a particular drug (i.e. after its introduction), or shows substantial deterioration of existing compatible symptoms and features. Since these ADRs can be hard to distinguish from the clinical picture associated with sarcoidosis, they are rather underestimated as serious causative agents of these drug-induced (DI) conditions. It is important to identify these ADRs because they bear on patient prognosis and treatment management [55,56].

By way of example we report the case of a 35-year-old male sarcoidosis patient known at our outpatient clinic, presenting with clinical deterioration. Initially, he presented to his general practitioner with fatigue, malaise, exercise limitation, and substantial depressive symptoms. As there was no indication for sarcoidosis treatment, his general practitioner started an antidepressant, viz. venlafaxine (75 mg daily). His drug history included no other medication. One month after the initiation of this treatment, he visited our outpatient clinic with progression of his complaints and agitation, having experienced no therapeutic effect of the venlafaxine. Clinical investigation excluded progression of his sarcoidosis. This prompted us to genotype three CYP genes (*CYP2C9*, *CYP2C19*, and *CYP2D6*) to investigate whether venlafaxine could be the cause of the clinical deterioration. It appeared that the patient was a poor metabolizer of *CYP2D6*, the most important phase I enzyme to metabolize venlafaxine. Discontinuation of this drug resulted in reduction of the symptoms.

It is a well known fact that there is interindividual variation in the metabolic processing of drugs. As was mentioned above, host factors, including genetic polymorphisms of CYP genes, may be important determinants of susceptibility to ADRs [57,58]. Increased plasma concentrations of venlafaxine can arise not only after an overdose of the drug but also in case of decreased clearance or even no metabolization, as was the case with our patient. Administering venlafaxine to poor metabolizers, as in our patient, places them at risk of accumulation of the drug to toxic concentrations [42,59]. ADRs may also occur if a drug inhibits a CYP isoenzyme and/or disrupts enzyme function in combination with other prescribed drugs, thus for instance causing an intermediate metabolizer genotype to phenotypically present as a poor metabolizer. Furthermore, genetic differences affecting the function of CYP enzymes may result in related changes in drug clearance and even the production of oxygen species. The above case highlights the potential benefit of both clinical and genetic risk stratification (PGx) prior to treatment. Moreover, early recognition is very important for clinicians, as drug cessation will significantly reduce the risk of ADR progression. In this case the problem was even more challenging, as the symptoms were compatible with sarcoidosis progression. Moreover, it illustrated that understanding the mechanisms of drug metabolism and interactions can help to prevent ADRs at an early stage.

## FUTURE DIRECTIONS

A pivotal aspect of sarcoidosis management is to regularly consider how to reduce the ADRs of GCs. Besides reducing GC doses by combining them with immunosuppressive drugs, new strategies to optimize treatment need to be explored, including lifestyle changes, physical therapy, and dietary guidance. For instance, a flavonoid-rich diet in conjunction with GCs has been reported to increase the efficacy of the GC therapy, thereby reducing the dose required for anti-inflammatory effect [60,61]. The benefits of flavonoids and other antioxidant supplements may be related to the restoration of normal antioxidant levels in patients with sarcoidosis [60].

PGx may lead to a more accurate drug management regime aimed at preventing unnecessary ADRs, as well as increasing the efficacy of the drug, and improving QoL [57]. There is a need to develop trials investigating the role of genetic variations not only in disease susceptibility and predicting prognosis, but also in treatment response, and in tailoring drug treatment to individual patients. Such investigations can help bridge the gap between ‘personalized’ and ‘evidence-based’ medicine. Improved recognition and a high index of suspicion are key to a proper diagnosis of ADRs and prompt withdrawal of the offending drug. Awareness of these DI disorders can play a vital role in preventing lengthy immunosuppressive therapies, as the main goal of management is drug cessation.

Polypharmacy is a growing iatrogenic condition precipitated by both medical necessity and external pressures. While the intention is to improve a patient's QoL and longevity, there are potential ADRs associated with unnecessary and inappropriate use of medicines. The indirect consequences of polypharmacy include exacerbation of drug-drug interactions, ADRs, increased likelihood of prescribing cascades, chronic dependence, and hospitalizations - all of which carry a significant health and economic burden. This highlights the need for a new systematic approach for fine-tuning the prescribing of medications. Improving communication between patients and physicians and medication list reviews by clinical pharmacists represent two practical approaches for preventing polypharmacy. The use of PGx testing constitutes a more plausible, empirical approach to polypharmacy, with the potential to alleviate both the economic burden and ADRs [62].

## CONCLUSION

A generally accepted approach to the management of sarcoidosis involves disease monitoring rather than active treatment with drugs in cases where the symptoms are tolerable and the risk of serious organ dysfunction is low. In addition, it is important to carefully evaluate the treatment of choice, considering its possible benefits and drawbacks. Moreover, one should consider patient-specific factors influencing the decisions. For instance, GCs should be avoided for those who are overweight and/or have diabetes, MTX should be avoided for those with chronic kidney disease, and AZA should be avoided for those with TPMT deficiency. In addition, DI damage should be considered, especially if the clinical situation deteriorates after the introduction of a particular drug in patients with sarcoidosis. Patients should be closely monitored for the development of potential ADRs or DI comorbidities. One should consider reducing the dose of immunosuppressive medications, as the risks of ADRs accumulate over time and in proportion to the cumulative dose.

The variability of drug response among patients, including those with sarcoidosis, is multifactorial. Therefore, improved recognition and a high index of suspicion are required for a proper diagnosis and timely withdrawal of the offending drug, as drug cessation is the mainstay of management. One should realize that polypharmacy by itself may also have a huge influence. However, early recognition and management of DI morbidity is a real clinical challenge. There is a need for a tailored approach that can be adopted in clinical practice, based on disease severity and risk profiles [57]. Trials should be developed to investigate the role of genetic variations not only in disease susceptibility and prognosis, but also in treatment response, and in tailoring drug treatment to individual patients.

## Acknowledgements

*The authors wish to thank the ild care foundation for facilitating the writing process.*

### Financial support and sponsorship

*None.*

### Conflicts of interest

*There are no conflicts of interest.*

## REFERENCES AND RECOMMENDED READING

Papers of particular interest, published within the annual period of review, have been highlighted as:

▪ of special interest
▪▪ of outstanding interest
